# Synergistic inhibition of *Haemonchus contortus* exsheathment by flavonoid monomers and condensed tannins

**DOI:** 10.1016/j.ijpddr.2015.06.001

**Published:** 2015-07-03

**Authors:** Chaweewan Klongsiriwet, Jessica Quijada, Andrew R. Williams, Irene Mueller-Harvey, Elizabeth M. Williamson, Hervé Hoste

**Affiliations:** a Chemistry and Biochemistry Laboratory, School of Agriculture, Policy and Development, University of Reading, P O Box 236, Reading RG6 6AT, UK; b UMR IHAP 1225 INRA/ENVT, Ecole Nationale Veterinaire de Toulouse, 31076, 23 Chemin des Capelles, Toulouse Cedex, France; c Faculty of Health and Medical Sciences, University of Copenhagen, 1871 Frederiksberg C, Denmark; d School of Pharmacy, P O Box 226, Whiteknights, Reading RG6 6AP, UK

**Keywords:** Nematodes, Procyanidins, Prodelphinidins, Proanthocyanidins, Mean degree of polymerisation, Flavan-3-ols, Quercetin, Luteolin

## Abstract

This study investigated the separate and combined anthelmintic (AH) effects of different phenolic compounds, including condensed tannins and flavonoids, all of which are known to occur in willow leaves, a potentially valuable dry season feed. A range of contrasting model tannins, which span the whole range of willow tannins, were isolated from tilia flowers, goat willow leaves, black currant leaves and red currant leaves. All together, the tested compounds represented the major tannin types (procyanidins and prodelphinidins) and flavonoid types (flavonols, flavones and flavanones). The larval exsheathment inhibition assay (LEIA) was used to assess their *in vitro* effects on *Haemonchus contortus* third stage larvae. Arbutin, vanillic acid, and taxifolin proved to be ineffective whereas naringenin, quercetin and luteolin were highly effective at 250 μM concentrations. Procyanidin (PC) tannins tended to be less active than prodelphinidin tannins (PD). Experiments with combinations of tannins and quercetin or luteolin revealed for the first time the existence of synergistic AH effects between tannins and flavonoid monomers. They also provided evidence that synergistic effects appear to occur at slightly lower concentrations of PC than PD. This suggests that the AH activity of condensed tannins can be significantly enhanced by the addition of quercetin or luteolin. This information may prove useful for plant breeding or selection and for designing optimal feed mixtures.

## Highlights

• Tannins and flavonoids showed anti-parasitic effects against *Haemonchus contortus*.
• This is the first report of their synergistic effects on larval exsheathment.
• Flavonoids enhanced especially the anthelmintic activities of procyanidin tannins.

## Graphical abstract

**Figure:**
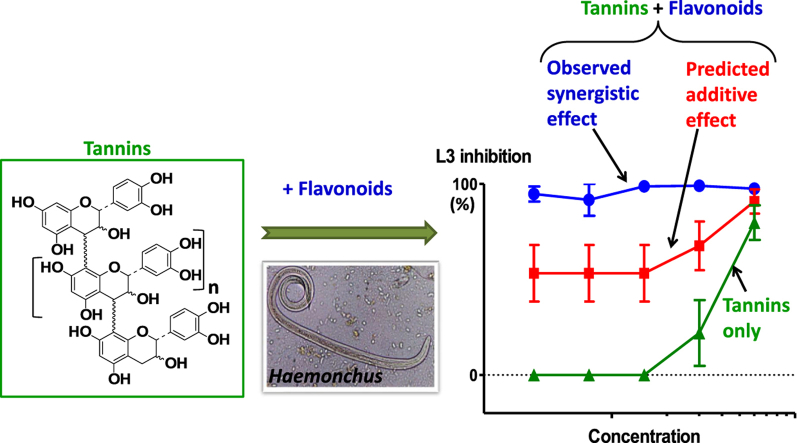

## 1 Introduction

Infection of small ruminants with gastrointestinal nematodes (GINs) remains a serious pathological problem across the world affecting animal health, welfare and production, as there is now a critically high level of drug resistance (Jackson et al., 2012). These GINs are able to develop resistance to new synthetic (anthelmintic, AH) drugs within a few years (Waller, 2006) and, therefore, sustainable livestock farming can no longer rely on deworming with AH drugs. New approaches for the sustainable control of GIN seek to lower parasite numbers to a manageable level or to modify their biological development and life cycle, rather than to eliminate them completely. Medicinal plants have been used since antiquity to treat worm infections (Hrckova and Velebny, 2013) and anti-parasitic properties of plants against GIN have been linked to the presence of proteinases (Stepek et al., 2004) and other secondary plant metabolites. Plant phenolics, flavonoids and condensed tannins have received considerable attention over the last few years (Barrau et al., 2005; Hoste et al., 2006, 2012) as plants that produce these compounds grow in all regions of the world. Condensed tannins (CT; *syn*. proanthocyanidins) can have direct and indirect AH effects (Min et al., 2003; Hoste et al., 2012), as well as having the potential to enhance the host's innate immune response (Tibe et al., 2012). The hypothesis for direct effects of polyphenolic compounds has been substantiated by several *in vitro* assays against different life-cycle stages (Bahuaud et al., 2006; Novobilský et al., 2011).

**Fig. 1:**
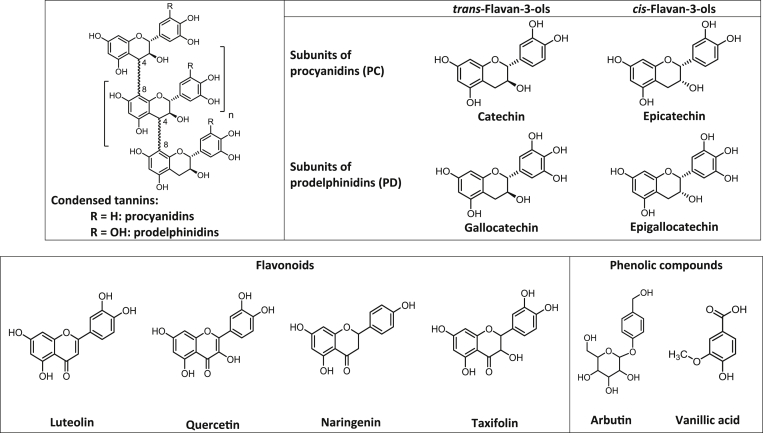
Structures of condensed tannins, flavonoids (flavan-3-ols, luteolin, quercetin, naringenin and taxifolin) and other polyphenols (arbutin and vanillic acid).

Condensed tannins are oligomers or polymers of flavan-3-ols and are classified into different subgroups. The two major CT types are procyanidins (PC), which have two OH-groups, and prodelphinidins (PD), which have three OH-groups in the B-ring (Fig. 1). The stereochemistry at the heterocyclic C-ring gives rise to either 2–3 *cis-* or 2–3 *trans-*flavan-3-ols and the average polymer length of CT mixtures is described in terms of mean degree of polymerisation (mDP). It is important to note that most condensed tannin-containing forages tend to contain complex mixtures of PC and PD homo- and hetero-polymers that are difficult to separate. However, a few plants specialise in the synthesis of either PCs or PDs and can thus serve as valuable sources for the different tannin types and as research tools for probing structure–activity relationships.

Since tannins and other flavonoids co-occur in plants, it is pertinent to investigate their combined potencies. To this end, we selected polyphenols, which are known to occur in willow (*Salix* spp, Salicaceae), as this species represents a potentially valuable nutraceutical forage (Moore et al., 2003; Diaz Lira et al., 2008; Mupeyo et al., 2011). Moreover, breeding programmes are currently focussing on increasing the *Salix* wood production as a renewable source of energy (Karp et al., 2011) and, therefore, it is timely to explore, which phenolic compositions can contribute most to the AH properties of the leaf by-products. The phenolic composition of willow leaves has been studied already by several groups (Jarrett and Williams, 1967; Pohjamo et al., 2003; Enayat and Banerjee, 2009) and we recently reported that willow tannins cover a wide range of PC/PD ratios (from 15/85 to 98/2) and *cis*/*trans* flavan-3-ol ratios (from 2/98 to 81/19) (Falchero et al., 2011). We now describe the isolation of distinct PC and PD types from specialist CT plant sources in order to cover the full spectrum of *Salix* tannins and tested their AH effects in the presence of commercially available phenolic monomers that are known to be present in *Salix* leaves. This allowed us to test two hypotheses; the first stated that CT and other naturally occurring polyphenolic monomers exert synergistic inhibitory effects on the *in vitro* L3 exsheathment of *Haemonchus contortus* and the second that these AH interactions depend on CT types.

## 2 Materials and methods

### 2.1 Chemicals and plant materials

Arbutin, luteolin and taxifolin were purchased from Apin Chemicals Ltd (Abingdon, U.K.); vanillic acid and naringenin from Alfa Aesar (Blackpool, U.K.); gallocatechin and quercetin from Sigma–Aldrich (Gillingham, U.K.); phosphate buffered saline (PBS) from bioMerieux (Lyon, France), Milton solution, sodium hypochlorite 2% w/v and sodium chloride 16.5% w/v from Milton (Inibsa laboratorios, Barcelona, Spain). Goat willow (*Salix caprea*), black currant (*Ribes nigrum*) and red currant (*Ribes rubrum*) leaves were collected in July and August 2012. Tilia (Linden) flowers (*Tilia* × *europaea*) were purchased from Flos (Mokrsko, Poland). These plant samples were chosen as they allowed the testing of different tannin types, all of which are known to occur in willow.

### 2.2 Preparation of plant extracts and tannin fractions

Freeze-dried, powdered plant material (25 g of each species) was extracted with an acetone:water mixture (7:3, v/v; 300 ml) (Williams et al., 2014a) and filtered. The filtrate was extracted with dichloromethane (250 ml) and the organic phase discarded. The aqueous phase was rotary evaporated under vacuum at 40 °C to remove residual organic solvents and then lyophilised. The freeze-dried extract was re-dissolved in distilled water, filtered under vacuum to remove insoluble particles and applied to a Sephadex LH-20 column. Fraction 1 was eluted with acetone/water (3:7, v/v) and fraction 2 with acetone/water (1:1, v/v). Acetone was removed in a rotary evaporator and the aqueous residue freeze-dried. Tannins were quantified and characterized by thiolysis with benzylmercaptan (Gea et al., 2011). Tannin composition was determined in terms of the percentage of PCs and PDs, *cis*- and *trans-*flavan-3-ol subunits and mean degree of polymerisation (mDP), which were calculated according to Gea et al. (2011).

### 2.3 Bioassays

#### 2.3.1 Haemonchus contortus strains

Third-stage larvae (L3) were obtained from sheep or goats infected with two monospecific strains of *H. contortus* that were susceptible to AH drugs (Alonso-Díaz et al., 2008). The Juan strain was obtained from donor sheep, which were kept indoors and infected monospecifically with *H. contortus*, and the larvae had been maintained in the laboratory for four months. This strain was used initially to investigate the effects of four F1- and four F2-tannin fractions and of several phenolic compounds. The INRA strain was obtained from monospecifically infected goats and the larvae had been maintained in the laboratory for 1 month before use. This strain was used to repeat experiments with the F2 tannin fractions, two of the flavonoids (quercetin and luteolin) and also with the tannin-flavonoid mixtures. The facilities hosting the animals and the trial was performed according to French ethical and welfare rules (agreement number C 31 555 27 of 19 August 2010).

#### 2.3.2 Larval exsheathment inhibition assay (LEIA)

The LEIA was performed as described previously (Bahuaud et al., 2006). Briefly, third-stage larvae were incubated with the tannin or flavonoid test solutions at 23 °C for 3 h, washed with PBS and centrifuged 3 times. The pelleted larvae were suspended in 200 μl water (ca 360–420 nematode L3 larvae) and 40 μl were removed to count the proportion of exsheathed larvae at 0 min. The remaining larvae (160 μl) were then subjected to the artificial exsheathment process by adding 40 μl of a Milton solution (2% w/v sodium hypochlorite and 16.5% sodium chloride) diluted in PBS, where the Milton:PBS ratio varied from 1 in 250 to 1 in 330 (this ratio was tested at the start of each experiment per batch in order to ensure 100% exsheathment within 60 min in the negative control). The numbers of ensheathed and exsheathed larvae were counted under a microscope at 0, 20, 40 and 60 min after contact with the Milton solution to investigate the proportion of exsheathed larvae over time. The tannin fractions were prepared as final serial concentrations of 600, 300, 150, 75, 37.5 μg fractions/ml and all other phenolic compounds at 1000, 500, 250, 100, 60 μM in PBS (0.1 M phosphate, 0.05 M NaCl, pH 7.2). The experiments with tannin-flavonoid mixtures were conducted using mixed solutions containing the same final concentrations of F2 fractions (600, 300, 150, 75, 37.5 μg fractions/ml) and combined with a fixed flavonoid concentration (either 60 or 30 μM quercetin or luteolin) in PBS. Four replicates were performed for each test solution and the negative control, L3 in PBS, was run in parallel.

### 2.4 Statistical analysis

The 50% effective concentration (EC_50_-value) was calculated using PoloPlus^©^ (version 1.0; LeOra Software Company, Petaluma, California, USA). These EC_50_-values are reported on the basis of tannin content in the F1 and F2 fractions (see Table 1). To assess possible synergistic effects of the tannin/flavonoid combinations, we used the observed inhibitory effects of the individual treatments to calculate the additive inhibitory effect that would be predicted to be achieved by the mixture under the assumption of independent action, using the definition of Bliss additivity (Bliss, 1939). The observed effect of the mixture was then compared to this predicted additive effect, with deviations from the predicted values indicating either synergy (greater effect) or antagonism (lower effect) (Williams et al., 2012).

## 3 Results

### 3.1 Condensed tannin analysis

The tannin fractions from the four plant species differed significantly in composition as shown in Table 1. The CT contents ranged from 43.4 to 99.8 g CT/100 g fraction; the mean degree of polymerisation (mDP) from 2.3 to 19.0; the tannin PC/PD ratios from 5.1/94.9 to 100/0 and the *cis/trans* flavan-3-ol ratios from 6.0/94.0 to 95.4/4.6. The fractions that eluted early from the Sephadex LH-20 column (F1 fractions) had lower CT contents (43.4–70.4 g CT/100 g fraction) and mDP-values (2.3–5.4) than the later eluting F2 fractions (CT-contents were 88.9–99.8 g CT/100 g fraction and mDP values were 6.4–19.0). Goat willow leaf and tilia flower tannins were mostly PCs, but black and red currant leaf tannins were mostly PDs. Stereochemistry added an additional distinguishing feature: red currant and tilia tannins contained predominantly *cis*-flavan-3-ols, but black currant and goat willow tannins had mostly *trans*-flavan-3-ols.

### 3.2 In vitro larval exsheathment inhibition by condensed tannins and other phenolic compounds

All tannin samples were first screened with four-month old larvae (Juan *H. contortus* strain from sheep) in the *in vitro* larval exsheathment inhibition assay. This experiment was then repeated with one-month old larvae (INRA *H. contortus* strain maintained on goats) and focussed only on the more potent F2 fractions. Results with the Juan strain gave 1.7–4.6-fold lower EC_50_-values than with the INRA strain (Table 2). Further experiments will be necessary to elucidate the underlying causes of this difference, such as larval age or origin, as older larvae from sheep were more sensitive to CTs (P < 0.05) than younger larvae from goats.

**Fig. 2:**
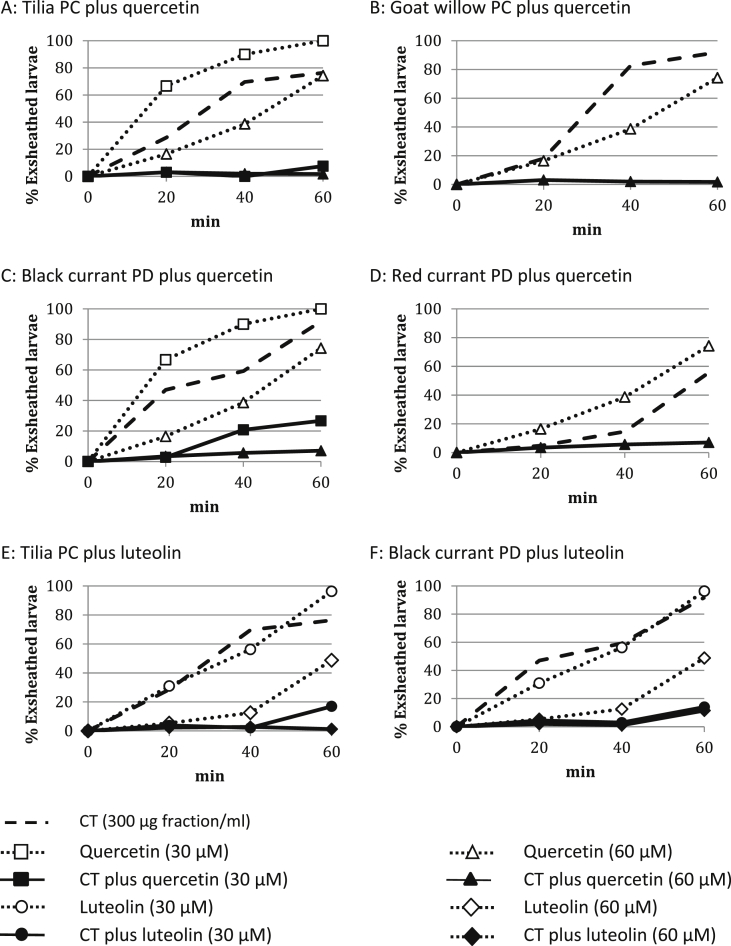
Larval exsheathment of *Haemonchus contortus* in the presence of different condensed tannin types (at 300 μg fraction/ml), which were isolated from tilia flowers, goat willow leaves, black currant leaves and red currant leaves, and quercetin or luteolin (30 and 60 μM) and their combinations (CT = condensed tannins; PC = procyanidin tannins; PD = prodelphinidin tannins).

These initial studies with the Juan strain showed that the EC_50_-values were generally lower with the F2 tannins than the corresponding F1 fractions from each plant source (Table 2). The Table and Fig. 2 also show that the PDs from black and red (in the web version) currant leaves tended to be more active than the PCs from tilia flowers and goat willow leaves.

The phenolic monomers differed greatly in their ability to inhibit larval exsheathment: arbutin, vanillic acid and taxifolin had no activity, whereas gallocatechin showed some activity at 1000 μM. However, naringenin, quercetin and luteolin were highly effective at 250 μM. Quercetin and luteolin were chosen (at 30 and 60 μM) for further investigation in combination with the different CTs.

### 3.3 In vitro larval exsheathment inhibition by condensed tannin-quercetin mixtures

Preliminary experiments using mixtures of serial CT concentrations with 200 or 100 μM quercetin completely inhibited exsheathment. Therefore, much lower quercetin concentrations (60 and 30 μM) were tested in the mixture experiments. The EC_50_-values of the mixture experiments with CTs and quercetin (30 and 60 μM) are shown in Table 2. The mixtures of PC fractions with the high quercetin concentration (60 μM) gave 3.8–5.7 fold lower EC_50_-values than these tannin fractions on their own; at the lower quercetin concentration (30 μM) the mixture gave a 2.3 fold lower EC_50_-value than the tilia tannin fraction. However, the mixtures of PD fractions with either quercetin concentration gave only 1.6–2.0 fold lower EC_50_-values than tannin fractions on their own.

The inhibition profiles generated by the mixtures revealed a dose–response relationship between *H. contortus* exsheathment and CT or quercetin concentrations as illustrated for the red currant PDs and quercetin (Supplementary Fig. 1). Mixed solutions containing low CT concentrations (37.5 and 75 μg of fractions/ml) combined with either quercetin concentration (30 or 60 μM) gave inhibition profiles that were similar to the quercetin solutions on their own (e.g. Supplementary Fig. 1A and B). However, mixed solutions with higher CT concentrations (300 and 600 μg of fractions/ml) and either quercetin concentration achieved > 90% inhibition. Quercetin with 150 μg of CT fractions/ml gave intermediate profiles (e.g. Supplementary Fig. 1C). Fig. 2 depicts the separate effects of each of the PC and PD fractions (at 300 μg of fraction/ml) on exsheathment and two quercetin or luteolin concentrations (30 and 60 μM). It also shows the combined effects of the mixed CT + flavonoid solutions. Tilia and goat willow PCs gave very similar inhibition profiles (Fig. 2A and B) whether on their own or mixed with quercetin. Red currant PDs, however, were more potent on their own than the black currant PDs (Fig. 2C and D). Tilia PCs were less active than black currant PDs on their own, but in the presence of flavonoids, all tilia PCs proved more active than the PD + flavonoid mixtures; this difference was significant (*P* < 0.05) for the luteolin combinations at 30 μM (see next section).

### 3.4 In vitro larval exsheathment inhibition by condensed tannin-luteolin mixtures

Experiments were also performed with CT + luteolin mixtures utilising the PC tannins from tilia flowers and PD tannins from black currant leaves. The EC_50_-values of these experiments are shown in Table 2. The mixture of tilia PC tannins with the higher luteolin concentration (60 μM) completely inhibited exsheathment so that the EC_50_-value could not be calculated. However, with the lower luteolin concentration (30 μM), the mixture gave a 4.7 fold lower EC_50_-value than the PC fraction on its own (75.9 vs 356 μg/ml). In comparison, the mixtures of black currant PDs with high (60 μM) and low (30 μM) luteolin concentrations were 8.9 and 1.6 fold more active than the PDs on their own (EC_50_-values of 31.2 and 170 vs 277 μg/ml). Fig. 2E depicts the separate effects of the tilia PCs and luteolin, and also the combined effects of the CT + luteolin mixtures. Luteolin at 30 μM produced a similar inhibition as the PCs on their own (300 μg fraction/ml), but luteolin at 60 μM was much more active.

Mixed solutions of lower CT concentrations (37.5, 75 and 150 μg fraction/ml) with the low luteolin (30 μM) concentration gave inhibition profiles similar to luteolin alone (data not shown), whereas mixed solutions at higher CT concentrations (300 and 600 μg fraction/ml) achieved > 95% inhibition (see Fig. 2E and F for the profiles at 300 μg fraction/ml). In contrast, combinations of all CT concentrations with luteolin at 60 μM gave generally greater than 95% inhibition (Fig. 2E and F give two examples). The tilia PC + luteolin and the black currant PD + luteolin mixtures (at 30 and 60 μM) gave similar inhibition patterns (Fig. 2E and F); however, there is some evidence that the mixtures with 60 μM luteolin and low PD concentrations (37.5, 75 and 150 μg fraction/ml) were not as effective as the corresponding PC mixtures (see also Fig. 4A and B).

### 3.5 Synergistic effects between condensed tannins and quercetin

**Fig. 3:**
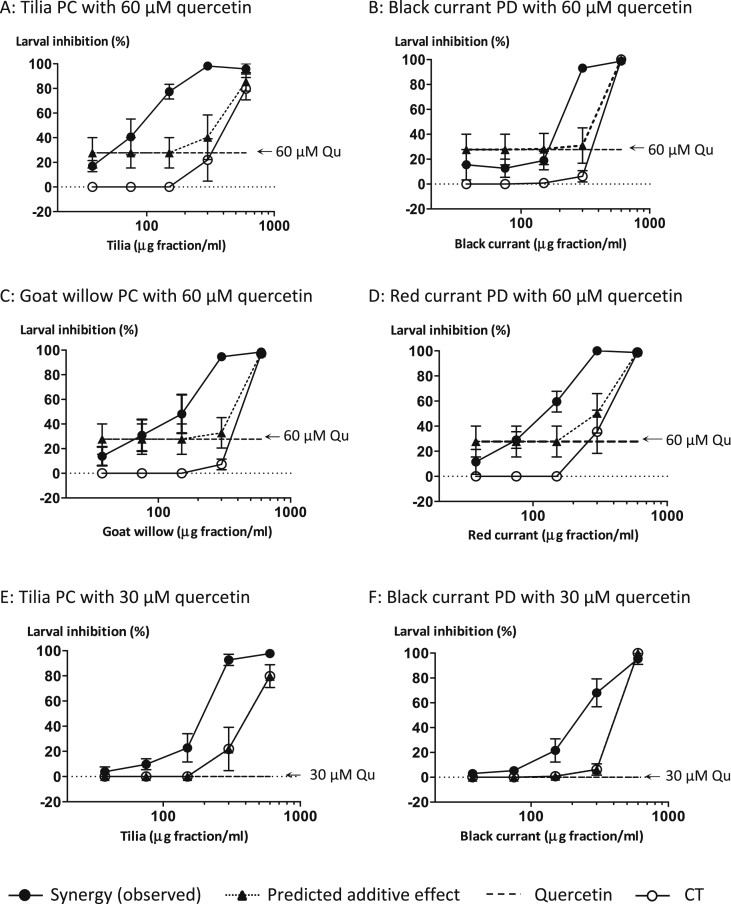
Synergistic effects between condensed tannins (CT) and quercetin (60 and 30 μM) on the inhibition of *Haemonchus contortus* larval exsheathment after 60 min.

The CT-quercetin mixtures revealed synergistic effects with CTs. Fig. 3 shows that the observed inhibition was much greater than the predicted additive inhibition calculated from the separate CT and quercetin data points (i.e. predicted additive effect): synergy was demonstrated between quercetin and tilia PCs (Fig. 3A) at 150 and 300 μg fraction/ml after 60 min, and with goat willow PCs (Fig. 3C) at 300 μg fraction/ml. Synergy was also observed with black currant PDs at 300 μg fraction/ml (Fig. 3B) and red currant PDs at 150 and 300 μg fraction/ml (Fig. 3D). In addition, synergy could be detected at 40 min but at a slightly lower level (not shown).

### 3.6 Synergistic effects between condensed tannins and luteolin

**Fig. 4:**
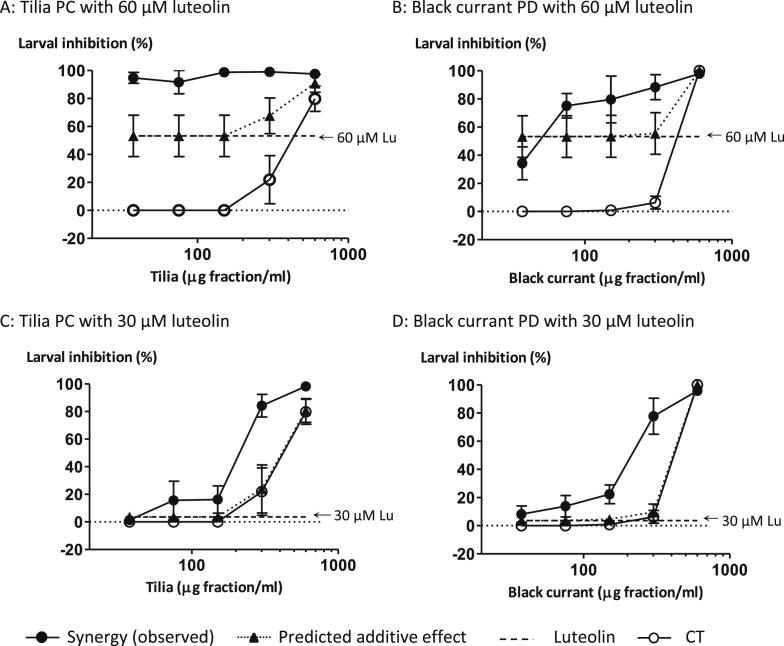
Synergistic effects between condensed tannins (CT) and luteolin (60 and 30 μM) on the inhibition of *Haemonchus contortus* larval exsheathment after 60 min.

Two of the slightly weaker AH tannins, tilia PCs and black currant PDs, were also evaluated for synergy at two luteolin concentrations. Fig. 4 shows the effects after 60 min. In the presence of 60 μM luteolin, tilia PCs exhibited clear synergy at all concentrations, but black currant PDs showed synergy only at 300 μg fraction/ml. At the lower luteolin concentration (30 μM), tilia PCs acted synergistically at 300 μg fraction/ml and black currant PDs at 150 and 300 μg fraction/ml.

## 4 Discussion

We chose the LEIA as it has advantages over other bioassays in that it is sensitive, reproducible and relevant to processes that occur *in vivo* (Alonso-Diaz et al., 2011; Azando et al., 2011). This assay was, therefore, chosen for evaluating the different CTs and flavonoids. The main aim was to investigate their combined effects as tannin-containing plants usually contain mixtures of CTs and other flavonoids.

PCs and PDs are the most common condensed tannins in forages and browse plants (Marais et al., 2000; Tibe et al., 2011) and tend to occur as complex combinations of homo- and hetero-polymers (Stringano et al., 2012) that are difficult to separate. However, well-defined tannin types are needed for unravelling the relationships between tannin structures and AH activities. A more straightforward approach, therefore, is to exploit plants that specialise in producing relatively pure but different tannin types (Williams et al., 2014a). The ultimate aim of this strategy is to provide plant breeders with targets for enhancing the nutraceutical properties of new animal feeds or varieties. Thus we isolated tannins as relatively pure homopolymers of 95.7–100% PCs and 89.9–94.7% PDs (Table 1) and also obtained two procyanidin subsets and two prodelphinidin subsets with low and high *cis/trans* flavan-3-ol ratios. Quercetin and luteolin are widely distributed in plants (Yang et al., 2008; López-Lázaro, 2009) and represent two important flavonoid subgroups, i.e. flavonols and flavones, respectively (Fig. 1), and were thus chosen for the combination experiments with CTs. Initial studies tested the AH effects of the four tannin types at several concentrations. Whilst the results confirmed that PDs tended to be better anthelmintics than PCs (Min and Hart, 2003; Brunet and Hoste, 2006; Novobilský et al., 2011), little is known about whether stereochemistry of the flavan-3-ol subunits has any effect. Our data provided an indication that the *cis/trans* stereochemistry of the flavan-3-ol subunits did not impact on AH activities (Table 1) in agreement with a previous study (Williams et al., 2014a), although further work will be needed as the average polymer sizes also varied for the PD subset (mDP-values were 10 and 19).

The data showed that tannin concentrations, which ranged from 43.4 to 99.8 g CT/100 g fraction, were not correlated with EC_50_ values. This points to the possibility that ‘impurities’ in these fractions may have moderated tannin activities. Typical impurities in aqueous acetone extracts and partially purified tannin fractions tend to consist of low molecular weight phenolics and flavonoids (Tibe et al., 2011).

Given that we observed increased inhibition of exsheathment with a mixture of tannins and quercetin or luteolin, we next quantified whether this effect was additive or synergistic in nature. Synergy refers to phenomena where two or more agents together produce an effect greater than would be predicted from their individual contributions. There are various mathematical models available to assess synergy, all of which have advantages and disadvantages (Williamson, 2001). Using the Bliss model (Williams et al., 2012), we observed synergistic effects between all CTs and quercetin or luteolin in terms of AH activity during the exsheathment of 3rd stage *H. contortus* larvae (Figs. 3 and 4). These CTs were tested at serial concentrations of 37.5, 75, 150, 300, 600 μg fraction/ml, which are physiologically relevant as extractable tannins range from 350 to 900 μg/ml in the sheep gut (Terrill et al., 1994; Barrau et al., 2005). However, no such information was found for quercetin or luteolin. It would seem that the AH effects of tilia PCs were slightly more enhanced, especially at lower concentrations, by quercetin or luteolin than the AH effects of PDs. A likely explanation could be that PCs offer more scope for enhancement as they tend to have weaker AH activities than PDs (Brunet and Hoste, 2006; Novobliský et al., 2011).

The mechanism(s) of the observed synergistic actions are not yet known. Tannins have been shown to deform nematode surfaces (Hoste et al., 2006; Williams et al., 2014a, b). Interestingly, exposure to extracts from tannin-containing plants (which would also have contained flavonoids) caused degeneration of muscle cells, accumulation of electron-dense vesicles and marked intracellular disorganisation (Brunet et al., 2011). These authors speculated whether the lesions observed in the cuticle of ensheathed L3 larvae could have been due to accumulation of metabolic products, which in turn could have produced cellular toxicity by blocking the metabolic exchange with the environment. Quercetin is well known as an inhibitor of P-glycoproteins (P-gp), which play an important role in the transport of xenobiotics (Lespine et al., 2012). It is not known whether this activity also contributes to enhancing the anthelmintic effects of tannins.

To summarise, this study provided the first evidence of synergistic effects between condensed tannins and two common flavonoids, quercetin and luteolin, in terms of inhibiting the *in vitro* exsheathment of *H. concortus* L3 larvae. Some of the synergistic effects occurred at lower PC than PD concentrations, perhaps implying that the generally weaker AH activity of PCs can benefit more from the addition of quercetin. These findings suggest that opportunities should be investigated for increasing anthelmintic activity by mixing plant materials that contain condensed tannins and quercetin or luteolin flavonoids, or to select plants with enhanced tannin and quercetin or luteolin contents.

## Conflicts of interest

The authors declared that there is no conflict of interest.

## Figures and Tables

**Table 1:** Composition of condensed tannins (CT) isolated from tilia flowers and leaves of goat willow, black currant and red currant.

Plant source	CT fraction	CT-content (g CT/100 g fraction)	mDP^a^	PC^b^ %	PD^b^ %	*cis*^c^*%*	*trans*^c^*%*
Goat willow	F1	70.4 (±1.7)	2.26 (0.0)	96.2 (0.0)	3.8	6.0 (0.0)	94.0
	F2	97.3 (±2.9)	6.35 (0.1)	95.7 (0.0)	4.3	8.9 (0.2)	91.1
Tilia	F1	52.5 (±1.5)	2.89 (0.0)	100 (0.0)	0.0	88.6 (0.1)	11.4
	F2	90.8 (±10.6)	8.80 (0.0)	100 (0.0)	0.0	95.4 (0.1)	4.6
Black currant	F1	66.4 (±0.4)	2.78 (0.0)	7.1 (2.4)	92.9	22.0 (0.2)	78.0
	F2	99.8 (±2.4)	9.67 (0.1)	5.3 (0.0)	94.7	17.8 (0.1)	82.2
Red currant	F1	43.4 (±3.8)	5.41 (0.1)	10.1 (0.0)	89.9	62.5 (0.5)	37.5
	F2	88.9 (±11.9)	19.0 (0.3)	5.1 (0.0)	94.9	74.6 (0.2)	25.4

a mDP: mean degree of polymerisation.

b PC, PD: percentage of procyanidin or prodelphinidin tannins.

c *cis, trans*: percentage of *cis*- or *trans*-flavan-3-ols.

**Table 2:** The 50% effective concentrations of tannin fractions (F1 and F2) and two flavonoids when tested *in vitro* in the exsheathment inhibition assay of *Haemonchus contortus* third stage larvae with either the Juan or INRA strains and 50% effective concentrations of CT plus two flavonoids (EC_50_-values; μg of CT or flavonoid/ml) at 30 and 60 μM concentrations on *H. contortus* third stage larvae INRA strain.

Plant source or compound	CT fraction	EC_50_ values (μg/ml) (95% CI)					
		CT		CT plus quercetin		CT plus luteolin	
		Juan	INRA	60 μM	30 μM	60 μM	30 μM
**PC rich fractions**							
Goat willow	F1	299 (273–324)^Y^	–	–	–	–	–
	F2	176 (140–222)^r,W^	394 (366–423)^b,s,U^	103 (72.3–149)^a,C^	–	–	–
Tilia	F1	142 (109–199)^U,V^	–	–	–	–	–
	F2	140 (116–168)^r,U^	356 (298–423)^b,s,T^	62.4 (42.8–90.1)^a,A^	156 (81.6–338)^c,A^	<37.5	75.9 (47.5–116)^a,d,A^
**PD rich fractions**							
Black currant	F1	178 (162–196)^W,X^	–	–	–	–	–
	F2	60.1 (28.8–90.1)^r,R^	277 (234–338)^b,s,S^	137 (89.3–232)^a,D^	164 (129–210)^c,A^	31.2 (10.9–52.0)^d^	170 (129–230)^c,e,B^
Red currant	F1	95.3 (71.4–126)^T^	–	–	–	–	–
	F2	75.8 (54.3–99.0)^r,S^	126 (101–155)^b,s,R^	78.8 (56.7–110)^a,B^	–	–	–
**Luteolin**		<71.5	17.1 (13.8–20.2)	–	–	–	–
**Quercetin**		<75.5	21.0 (19.2–23.0)	–	–	–	–

^a,b,c,d,e,r,s,A,B,C,D,R,S,T,U,V,W,X^: Lower case letters are used to indicate significant differences within rows and upper case letters within columns (P < 0.05). ^r,s,R,S,T,U,V,W,X^ These letters indicate significant differences between the EC_50_-values of Juan and INRA strains (experiments are based on tannin fractions) (P < 0.05). ^a,b,c,d,A,B,C,D^ These letters indicate significant differences between EC-_50_ value of the tannin-flavonoid combination experiments with the INRA strain (P < 0.05).

## References

[bib1] Alonso-Díaz M.A., Torres-Acosta J.F.J., Sandoval-Castro C.A., Aguilar-Caballero A.J., Hoste H. (2008). *In vitro* larval migration and kinetics of exsheathment of *Haemonchus contortus* larvae exposed to four tropical tanniniferous plant extracts. Vet. Parasitol..

[bib2] Alonso-Díaz M.A., Torres-Acosta J.F.J., Sandoval-Castro C.A., Hoste H. (2011). Comparing the sensitivity of two *in vitro* assays to evaluate the anthelmintic activity of tropical tannin rich plant extracts against *Haemonchus contortus*. Vet. Parasitol..

[bib3] Azando E.V., Hounzangbe-Adote M.S., Olounlade P.A., Brunet S., Fabre N., Valentin A., Hoste H. (2011). Involvement of tannins and flavonoids in the *in vitro* effects of *Newbouldia laevis* and *Zanthoxylum zanthoxyloides* extracts on the exsheathment of third-stage infective larvae of gastrointestinal nematodes. Vet. Parasitol..

[bib4] Bahuaud D., Martinez-Ortiz de Montellano C., Chauveau S., Prevot F., Torres-Acosta F., Fouraste I., Hoste H. (2006). Effects of four tanniferous plant extracts on the *in vitro* exsheathment of third-stage larvae of parasitic nematodes. Parasitology.

[bib5] Barrau E., Fabre N., Fouraste I., Hoste H. (2005). Effect of bioactive compounds from sainfoin (*Onobrychis viciifolia*) on the *in vitro* larval migration of *Haemonchus contortus*: role of tannins and flavonol glycosides. Parasitology.

[bib6] Bliss C.I. (1939). The toxicity of poisons applied jointly. Ann. Appl. Biol..

[bib7] Brunet S., Hoste H. (2006). Monomers of condensed tannins affect the larval exsheathment of parasitic nematodes of ruminants. J. Agric. Food Chem..

[bib8] Brunet S., Fourquaux I., Hoste H. (2011). Ultrastructural changes in the third-stage, infective larvae of ruminant nematodes treated with sainfoin (*Onobrychis viciifolia*) extract. Parasitol. Int..

[bib9] Diaz Lira C.M., Barry T.N., Pomroy W.E., McWilliam E.L., Lopez-Villalobos N. (2008). Willow (*Salix* spp.) fodder blocks for growth and sustainable management of internal parasites in grazing lambs. Anim. Feed Sci. Tech..

[bib10] Enayat S., Banerjee S. (2009). Comparative antioxidant activity of extracts from leaves, bark and catkins of *Salix aegyptiaca* sp. Food Chem..

[bib11] Falchero L., Brown R.H., Karp A., Hanley S., Shield I., Mueller-Harvey I. (2011). The structural diversity of condensed tannins in willows (*Salix* spp.): a first screening to improve the nutritional quality of ruminant products. Adv. Anim. Biosci..

[bib12] Gea A., Stringano E., Brown R.H., Mueller-Harvey I. (2011). *In situ* analysis and structural elucidation of sainfoin (*Onobrychis viciifolia*) tannins for high-throughput germplasm screening. J. Agric. Food Chem..

[bib13] Hoste H., Jackson F., Athanasiadou S., Thamsborg S.M., Hoskin S.O. (2006). The effects of tannin-rich plants on parasitic nematodes in ruminants. Trends Parasitol..

[bib14] Hoste H., Martinez-Ortiz-De-Montellano C., Manolaraki F., Brunet S., Ojeda-Robertos N., Fourquaux I., Torres-Acosta J.F., Sandoval-Castro C.A. (2012). Direct and indirect effects of bioactive tannin-rich tropical and temperate legumes against nematode infections. Vet. Parasitol..

[bib15] Hrckova G., Velebny S., Hrckova G., Velebny S. (2013). Parasite helminths of humans and animals: health impact and control. Pharmacological Potential of Selected Natural Compounds in the Control of Parasitic Diseases.

[bib16] Jackson F., Varady M., Bartley D.J. (2012). Managing anthelmintic resistance in goats—Can we learn lessons from sheep?. Small Rumin. Res..

[bib17] Jarrett J.M., Williams A.H. (1967). The flavonoid glycosides of *Salix purpurea*. Phytochemistry.

[bib18] Karp A., Hanley S.J., Trybush S.O., Macalpine W., Pei M., Shield I. (2011). Genetic improvement of willow for bioenergy and biofuels. J. Integr. Plant Biol..

[bib19] Lespine A., Ménez C., Bourguinat C., Prichard R.K. (2012). P-glycoproteins and other multidrug resistance transporters in the pharmacology of anthelmintics: prospects for reversing transport-dependent anthelmintic resistance. Int. J. Parasitol. Drugs Drug Resist.

[bib20] López-Lázaro M. (2009). Distribution and biological activities of the flavonoid luteolin. Mini-Rev. Med. Chem..

[bib21] Marais J.P.J., Mueller-Harvey I., Brandt E.V., Ferreira D. (2000). Polyphenols, condensed tannins and other natural products in *Onobrychis viciifolia* (sainfoin). J. Agric. Food Chem..

[bib22] Min B.R., Barry T.N., Attwood G.T., McNabb W.C. (2003). The effect of condensed tannins on the nutrition and health of ruminants fed fresh temperate forages: a review. Anim. Feed Sci. Tech..

[bib23] Min B.R., Hart S.P. (2003). Tannins for suppression of internal parasites. J. Anim. Sci..

[bib24] Moore K.M., Barry T.N., Cameron P.N., Lopez-Villalobos N., Cameron D.J. (2003). Willow (*Salix* sp.) as a supplement for grazing cattle under drought conditions. Anim. Feed Sci. Tech..

[bib25] Mupeyo B., Barry T.N., Pomroy W.E., Ramírez-Restrepo C.A., López-Villalobos N., Pernthaner A. (2011). Effects of feeding willow (*Salix* spp.) upon death of established parasites and parasite fecundity. Anim. Feed Sci. Tech..

[bib26] Novobilský A., Mueller-Harvey I., Thamsborg S.M. (2011). Condensed tannins act against cattle nematodes. Vet. Parasitol..

[bib27] Pohjamo S.P., Hemming J.E., Willför S.M., Reunanen M.H.T., Holmbom B.R. (2003). Phenolic extractives in *Salix caprea* wood and knots. Phytochemistry.

[bib28] Stepek G., Behnke J.M., Buttle D.J., Duce I.R. (2004). Natural plant cysteine proteinases as anthelmintics. Trends Parasitol..

[bib29] Stringano E., Hayot Carbonero C., Smith L.M.J., Brown R.H., Mueller-Harvey I. (2012). Proanthocyanidin diversity in the EU ‘HealthyHay’ sainfoin (*Onobrychis viciifolia*) germplasm collection. Phytochemistry.

[bib30] Terrill T.H., Waghorn G.C., Woolley D.J., McNabb W.C., Barry T.N. (1994). Assay and digestion of 14C-labelled condensed tannins in the gastrointestinal tract of sheep. Br. J. Nutr..

[bib31] Tibe O., Meagher L.P., Fraser K., Harding D.R.K. (2011). Condensed tannins and flavonoids from the forage legume sulla (*Hedysarum coronarium*). J. Agric. Food Chem..

[bib32] Tibe O., Pernthaner A., Sutherland I., Lesperance L., Harding D. (2012). Condensed tannins from Botswanan forage plants are effective priming agents of γδ T cells in ruminants. Vet. Immunol. Immunopathol..

[bib33] Waller P.J. (2006). From discovery to development: current industry perspectives for the development of novel methods of helminth control in livestock. Vet. Parasitol..

[bib34] Williams A.R., Douglas A.D., Miura K., Illingworth J.J., Choudhary P., Murungi L.M., Furze J.M., Diouf A., Miotto O., Crosnier C., Wright G.J., Kwiatkowski D.P., Fairhurst R.M., Long C.A., Draper S.J. (2012). Enhancing blockade of *Plasmodium falciparum* erythrocyte invasion: assessing combinations of antibodies against PfRH5 and other merozoite antigens. PLoS Pathog..

[bib35] Williams A.R., Fryganas C., Ramsay A., Mueller-Harvey I., Thamsborg S.M. (2014). Direct anthelmintic effects of condensed tannins from diverse plant sources against *Ascaris suum*. PLoS ONE.

[bib36] Williams A.R., Ropiak H.M., Fryganas C., Desrues O., Mueller-Harvey I., Thamsborg S.M. (2014). Assessment of the anthelmintic activity of medicinal plant extracts and purified condensed tannins against free-living and parasitic stages of *Oesophagostomum dentatum*. Parasit. Vectors.

[bib37] Williamson E.M. (2001). Synergy and other interactions in phytomedicines. Phytomedicine.

[bib38] Yang R.-Y., Lin S., Kuo G. (2008). Content and distribution of flavonoids among 91 edible plant species. Asia Pac. J. Clin. Nutr..

