# Supplementary material for: Synergistic inhibition of Haemonchus contortus exsheathment by flavonoid monomers and condensed tannins
Source: Int J Parasitol Drugs Drug Resist. 2015 Jul 3;5(3):127–34. doi: 10.1016/j.ijpddr.2015.06.001 (PMC4506970; doi:10.1016/j.ijpddr.2015.06.001)
Supplement: Supplementary file 1 [file mmc1.docx]

**Supplementary material**

| A: Red currant CT at 37.5 µg fraction/ml | B: Red currant CT at 75 µg fraction/ml | |
| --- | --- | --- |
| C: Red currant CT at 150 µg fraction/ml | D: Red currant CT at 300 µg fraction/ml | |
| E: Red currant CT at 600 µg fraction/ml |  | Red currant CT  Quercetin (60 µM)  CT plus quercetin (60 µM) |

**Fig. 1.**
